# Bacterial Community Characteristics and Enzyme Activities in *Bothriochloa ischaemum* Litter Over Progressive Phytoremediation Years in a Copper Tailings Dam

**DOI:** 10.3389/fmicb.2020.565806

**Published:** 2020-12-21

**Authors:** Tong Jia, Yuwen Wang, Baofeng Chai

**Affiliations:** Shanxi Key Laboratory of Ecological Restoration on Loess Plateau, Institute of Loess Plateau, Shanxi University, Taiyuan, China

**Keywords:** copper tailings, *Bothriochloa ischaemum*, litter, bacterial community, enzyme activities

## Abstract

Litter decomposition is the key link between material circulation and energy flow in ecosystems, resulting from the activity of resident microbes and various enzymes. This study investigated enzyme activity in litter and associated microbial community characteristics to help clarify the internal mechanisms associated with litter decomposition, while also providing researchers a scientific basis for soil remediation in mining areas. Results confirmed that the nutrient content of *Bothriochloa ischaemum* litter significantly increased as phytoremediation years progressed, while enzyme activities in litter varied over different phytoremediation years. During the litter decomposition process, cellulase predominated in the early phytoremediation stage and catalase predominated in the intermediate phytoremediation stage. Obvious differences were found in bacterial community structure and diversity over progressive phytoremediation years. Predominant bacterial genera mainly included *Massilia*, *Sphingomonas*, *Curtobacterium*, *Amnibacterium*, and *Methylobacterium*. Moreover, *Methylorosula* and *Jatrophihabitans* had relatively higher betweenness centrality, and played important roles in bacterial community positive interactions. Additionally, total nitrogen (TN) and total zinc in soil, sucrase and catalase activity in litter were the main environmental factors that affected the structural framework of bacteria in *B. ischaemum* litter. However, TN had the greatest overall effect on the structural framework of bacteria in litter. Results from this study can help our understanding of the role that litter plays in degraded ecosystems. Our results also provide a scientific basis for improving poor quality soil in areas affected by copper tailings while also amending ecological restoration efficiency.

## Introduction

Litter is a crucial source of organic matter and nutrient elements in ecosystems, while litter decomposition plays an important role in ecosystem material circulation and energy flow (Song et al., 2014; Müller et al., 2016). Although litter decomposition is mainly driven by microorganisms (Crumsey et al., 2015), enzymes also play a critical role. Microorganisms affect litter decomposition by producing and optimizing enzymatic distribution (Ostaszewski and Nissen, 1988) while concurrently effecting soil properties. The production of extracellular enzymes depends on the type of microorganism and its nutritional requirements, and such production typically reflects the dynamics of litter degradation. In a study on forest litter in a northern subtropical latitude, urease and cellulase were found to contribute significantly to litter decomposition (Riemer et al., 1974). Changes in bacterial and fungal nutrient layers are also associated with specific enzyme activities (Li et al., 2019). Results from a previously published study determined that bacterial communities that undergo disparate litter nutrient transformation and humus decomposition processes lead to interspecific competition, thus altering bacterial community composition and diversity (Liu et al., 2017). The role that bacterial communities play in the transformation of organic substrates and the release of mineral elements is therefore indispensable (Yang et al., 2018). Additionally, many strains isolated from soil can degrade cellulose (Lopez-Mondejar et al., 2016). Accordingly, understanding characteristics of bacterial communities and associated enzyme activities in litter is crucial when exploring litter decomposition mechanisms.

The response of bacterial communities to environmental factors at different spatial scales remains controversial among researchers. Studies have shown that climate is the key factor for both the global and regional scale regulation of litter decomposition (Rastogi et al., 2012; Bradford et al., 2017). Nutrient content and nutrient availability also play important roles at a local scale (Allison and Vitousek, 2004). At present, research on litter has mainly focused on forest, grassland and other ecological systems, such as the impact of litter decomposition on soil nutrients and microbial communities (Xiangqin et al., 2011). However, studies related to microbial community characteristics and enzyme activities of litter in degraded ecosystems are relatively rare. Specifically, studies on litter phytoremediation in mining areas contaminated with heavy metals have yet to be seriously conducted. Consequently, this remediation approach in these specific areas remains largely unexplored and unresolved.

The Eighteen River tailings dam of the Northern Copper Mine is in Yuanqu County, Shanxi Province, China. In this tailings dam, large-scale mining activities have resulted in an enormous amount of tailings accumulation that has subsequently resulted in severe pollution and damage to the local ecological environment (Oka and Uchida, 2018). Heavy metal contamination in the soil of this copper tailings dam is severe and soil nutrition is poor. Thus, ecological restoration in this mining area is urgently required. *Bothriochloa ischaemum* is an important phytoremediation species used in copper tailings dams. This perennial herbaceous grass species tolerate warm seasonal temperatures while also being drought tolerant and highly regenerable (Qingan et al., 2006). As phytoremediation a progress, litter accumulates year by year, eventually becoming the primary organic matter source during the succession process of ore soil. Litter is the link between vegetation and soil interface, and it plays an important role in the function of aboveground and belowground ecosystems (Bani et al., 2018; Tan et al., 2020). Litter decomposition controls both the material and chemical cycles of terrestrial ecosystems (Cotrufo et al., 2015). Therefore, exploring litter decomposition characteristics and associated influencing factors in a copper tailing dam will help provide new insight into understanding the crucial role that litter decomposition plays in nutrient cycling in this region. In this study, we selected three sub-dams representative of different phytoremediation years as the sampling plots where *B. ischaemum* specimens were collected. We hypothesized that soil and litter properties, litter enzyme activities, as well as bacterial community characteristics varied over different phytoremediation years, while litter properties and enzyme activities rather than soil properties would drive litter bacterial community structures and functions. To test our hypothesis, we conducted this study (i) to compare characteristics of bacterial community structure and diversity in *B. ischaemum* litter during different phytoremediation years; (ii) to analyze dynamic changes of enzyme activities in *B. ischaemum* litter as phytoremediation progresses; (iii) and to explore the main driving factors that affect bacterial communities and enzyme activities in *B. ischaemum* litter during each recovery stage. By clarifying the relationship between the litter bacterial community and resident environmental factors during different phytoremediation years, the ecological role that litter plays in degraded ecosystems can be further understood. This study also offers a scientific basis for researchers to improve poor quality soil while enhancing the efficiency of ecological restoration in copper tailings areas.

## Materials and Methods

### Site Description and Soil Sampling

Construction of the Eighteen River tailings of the Northern Copper Mine (35°15′∼35°17′ N, 118°38′∼111°39′ E) in the southern region of the Zhongtiao Mountains started in 1969. This region is under the influence of a continental monsoon climate. The average annual temperature is 13.5°C, while the annual precipitation is 631 mm. Currently, the Eighteen River tailings dam is composed of 16 sub-dams (Jia et al., 2019). The main constituents of the dam comprise of copper tailings and artificial loess. The slope ratio of the dam is 1:6.

Three sub-dams (S523, S536, and S560) that have individually undergone 38, 22, and 5 phytoremediation years, respectively, were selected for sampling. The latitudinal and longitudinal coordinates are 35°26′N and 111°66′E. Top soil samples as well as litter from the soil surface were collected within the *B. ischaemum* distribution area of each sub-dam, where three replications were made for each sub-dam. Sampling plots were spaced at a distance of between 100 and 120 m. For each replicate, we collected 200 g of soil and 100 g of litter samples to measure chemical properties and enzyme activities. A total of 18 litter and soil samples were collected. Samples were sealed in self-sealing plastic bags and placed inside boxes containing ice before being immediately being transported to the lab. Litter samples were then subdivided into two, one stored at −20°C for high-throughput sequencing and the other stored at 4°C along with soil samples to determine physiochemical properties. Sterile gloves were worn throughout the sampling process to avoid sample contamination.

### Chemical Properties and Enzyme Activities of Samples

The drying method was used to determine soil water content (SWC). An elemental analyzer (vario EL/MACRO cube, Elementar, Hanau, Germany) was used to measure total soil carbon (TC) and total soil nitrogen (TN) content in litter (TC_Litter and TN_Litter, respectively). Soil pH was measured after shaking samples in a soil-water (1:2.5 m/v) suspension for 30 min (Wang et al., 2018). Soil particle size (PS) was measured by using a Mastersizer 3000 laser diffraction particle size analysis instrument (Malvern Co., Ltd., Malvern, United Kingdom). Heavy metal elements, including arsenic (As), cadmium (Cd), copper (Cu), lead (Pb), and zinc (Zn), were measured using Inductively Coupled Plasma-Atomic Emission Spectrometry (iCAP 6000, Thermo Fisher, United Kingdom). Potassium permanganate titration was used to measure catalase. 3,5-Dinitrosalicylic acid colorimetry was used to measure sucrase and cellulase, while phenol-sodium hypochlorite colorimetry was used to measure urease. Finally, iodimetry was used to measure polyphenol oxidase.

### Techniques Used for DNA Extraction, PCR Amplification and MiSeq Sequencing

Nine litter samples were initially washed three times in a sterile phosphate buffer solution (PBS: NaCl, KCl, Na_2_HPO_4_, and KH_2_PO_4_) before filtering through a sterile membrane filter (0.2 μm pore size) (Millipore, Jinteng, Tianjin, China). These filtered samples, used to extract microbial DNA, were then sealed in sterile centrifuge tubes. The E.Z.N.A.^®^ Soil DNA Kit (Omega Bio-Tek, Norcross, GA, United States) was employed for the extraction of microbial plant and soil DNA under the manufacturer’s protocol. The NanoDrop ND-1000 UV-Vis Spectrophotometer (NanoDrop Technologies, Wilmington, DE, United States) was used to quantify extracted DNA. Amplification of the V5–V7 hyper variable region of the 16S rRNA bacterial gene was conducted using primers 799F (5′-AACMGGATTAGATACCCKG-3′) and 1193R (5′-ACGTCATCCCCACCTTCC-3′). We conducted sequencing at Shanghai Majorbio Bio-pharm Technology (Shanghai, China), applying the MiSeq platform (Illumina, Inc., United States).

### Processing of Sequencing Data

The raw 16S rRNA gene sequencing reads were demultiplexed, quality-filtered by fastp version 0.20.0 (Chen et al., 2018) and merged by FLASH version 1.2.7 (Magoè and Salzberg, 2011) with the following criteria: (i) the 300 bp reads were truncated at any site receiving an average quality score of <20 over a 50 bp sliding window, and the truncated reads shorter than 50 bp were discarded, reads containing ambiguous characters were also discarded; (ii) only overlapping sequences longer than10 bp were assembled according to their overlapped sequence. The maximum mismatch ratio of overlap region is 0.2. Reads that could not be assembled were discarded; (iii) samples were distinguished according to the barcode and primers, and the sequence direction was adjusted, exact barcode matching, 2 nucleotide mismatch in primer matching. Operational taxonomic units (OTUs) with 97% similarity cutoff were clustered using UPARSE version 7.1, and chimeric sequences were identified and removed (Stackebrandt et al., 1994; Edgar, 2013). The taxonomy of each OTU representative sequence was analyzed by RDP Classifier version 2.2 (Wang et al., 2007) against the silva132/16s_bacteria database using confidence threshold of 0.7. The bacterial sequences were banked in the National Center for Biotechnology Information (NCBI) database under the Sequence Read Archive (SRA) accession: PRJNA625865.

### Statistical Analysis

Differences in chemical properties among soil, litter and enzyme activities of each sub-dam, which did not comply with the normal distribution, were tested using the Kruskal–Wallis *H* test in SPSS Statistics version 24.0. Analysis of the microbial community structure was performed using SPSS Statistics version 24.0 and SigmaPlot version 14.0. Spearman’s rank correlation coefficient was employed to analyze relationships among environmental factors and microbial community diversity correlation analysis, and Venn diagrams were generated using R3.5.3. Redundancy analysis (RDA) was conducted in Canoco 5.0 (Microcomputer Power, United States).

### Bioinformatics Analysis

Non-metric multidimensional scaling (NMDS) analysis was conducted on the bacterial community structure based on Bray–Curtis Dissimilarity, and ANOSIM was used to analyze inter-group differences. Additionally, variance inflation factor (VIF) analysis was used to eliminate the high collinearity of environmental factors using the “vegan package” in R 3.5.3. We used the interactive platform Gephi to explore and visualize networks (Bastian et al., 2009). phylogenetic investigation of communities by reconstruction of unobserved states (PICRUSt) program was used to predict bacterial community functions based on the KEGG database (Langille et al., 2013), which could obtain the correlated the microbial functional features with the important enzymes. This method analysis was performed using the free online platform of Majorbio Cloud Platform^1^.

## Results

### Soil and Litter Physical and Chemical Properties

The soil TC, TN, C/N, PS, and SWC were increased as phytoremediation years progressed. Moreover, soil TC in S523 was higher than in the other sub-dams dams (i.e., S523, S536, and S560) (Table 1). Litter TC, TN, and C/N varied among the three sub-dams. During the early phytoremediation stage, soil As and Cd concentrations were significantly lower than the other periods. In contrast, Cu content was significantly higher during the early phytoremediation stage (Table 1). No significant differences were found in Pb and Zn concentrations of all three sub-dams. Litter TC content in S536 was greater than that in S560. Litter TN content in S523 was higher than that in the other sub-dams (Table 1). Furthermore, TC and TN concentrations in litter were greater than a factor of 53.6 and 39.1, respectively, than that in soil (Table 1).

**TABLE 1:** Soil and litter properties over progressive phytoremediation years.

**Physical and chemical factors**		**S523**	**S536**	**S560**
Soil	TC_Soil (g/kg)	12.16 ± 1.67*a*	6.47 ± 1.95*b*	5.57 ± 2.07*b*
	TN_Soil (g/kg)	0.28 ± 0.13	0.24 ± 0.14	0.22 ± 0.13
	C/N_Soil	53.64 ± 7.00	30.35 ± 6.71	28.53 ± 31.00
	SWC (%)	0.09 ± 0.02	0.07 ± 0.03	0.06 ± 0.03
	pH	8.19 ± 0.08	8.11 ± 0.10	8.22 ± 0.16
	PS (μm)	49.03 ± 12.68	41.30 ± 10.92	36.07 ± 5.43
	As (mg/kg)	14.71 ± 1.53*ab*	25.44 ± 9.50*a*	4.58 ± 1.30*b*
	Cd (mg/kg)	6.38 ± 1.07*a*	7.58 ± 0.83*a*	3.19 ± 0.08*b*
	Cu (mg/kg)	378.18 ± 43.37*b*	347.03 ± 18.94*b*	487.84 ± 51.10*a*
	Pb (mg/kg)	243.14 ± 39.00	173.07 ± 37.91	185.81 ± 107.93
	Zn (mg/kg)	87.53 ± 23.77	72.36 ± 10.87	51.28 ± 15.02
Litter	TC_Litter (g/kg)	434.02 ± 0.88*ab*	442.24 ± 3.36*a*	420.80 ± 1.69*b*
	TN_Litter (g/kg)	13.28 ± 1.36*a*	4.72 ± 0.14*c*	10.91 ± 0.78*b*
	C/N_Litter	32.96 ± 3.49*c*	93.79 ± 3.15*a*	38.73 ± 2.68*b*

*mean total nitrogen (TN), total carbon (TC), the carbon and nitrogen ratio (C/N), soil water content (SWC), average particle size (PS), arsenic (As), cadmium (Cd), copper (Cu), lead (Pb) and zinc (Zn). Data are means ± standard deviation. Significant differences between sites (*P* < 0.05) are represented by letters (a > b > c).*

Five litter enzymes exhibited significant differences as phytoremediation years progressed (Table 2). Urease and sucrase in S523 were significantly higher than corresponding values in the other sub-dams. Catalase and polyphenol oxidase in litter of S536 were significantly higher than that corresponding values in S523. Cellulase activity decreased significantly as phytoremediation years progressed (Table 2), while urease activity increased as phytoremediation years progressed (Table 2).

**TABLE 2:** Enzyme activities in *Bothriochloa ischaemum* litter over different phytoremediation years.

**Enzyme activity**	**S523**	**S536**	**S560**
Urease [mg/(g⋅24 h)]	2.35 ± 0.42a	0.86 ± 0.38b	0.36 ± 0.31b
Sucrase [mg/(g⋅24 h)]	12.25 ± 1.22a	1.30 ± 0.19b	2.53 ± 1.00b
Catalase [mg/(g⋅20 min)]	1.78 ± 0.15b	4.02 ± 0.84a	2.16 ± 0.48ab
Cellulase [mg/(g⋅72 h)]	0.57 ± 0.09c	1.14 ± 0.12b	1.38 ± 0.07a
Polyphenol oxidase (mL/g)	4.80 ± 0.84b	5.70 ± 0.55a	5.20 ± 0.45ab

*Data are means ± standard deviation. Different letter cases indicate that the means are significantly different over different phytoremediation years. Significant differences between sites (*P* < 0.05) are represented by letters (a > b > c).*

### Composition and Diversity of Litter Bacterial Communities

**FIGURE 1:**
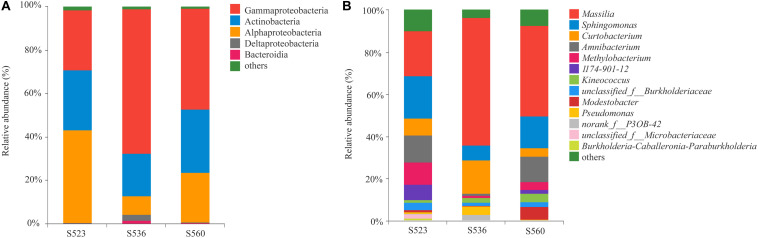
The relative abundance of the litter bacterial community at the class **(A)** and genus **(B)** level over different phytoremediation years.

**FIGURE 2:**
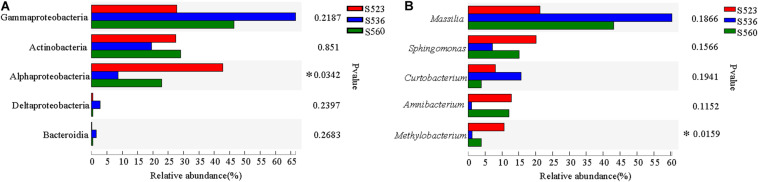
Differences in the dominant bacterium at the class **(A)** and genus **(B)** level over different phytoremediation years (*P* < 0.05).

Illumina high-throughput sequencing was used to analyze the structure and diversity of the *B. ischaemum* litter bacterial communities. A total of 297 OTUs were measured in the three samples, with a total of 15,4893 effective sequences and 5,821,3043 bp and an average length of 376 bp. The dominant litter bacteria were Gammaproteobacteria, Actinobacteria, Alphaproteobacteria, Deltaproteobacteria, and Bacteroidia (Figure 1A). At a genus level, the dominant bacteria were *Massilia*, *Sphingomonas*, *Curtobacterium*, *Amnibacterium*, and *Methylobacterium* (Figure 1B). The relative abundances of Alphaproteobacteria and *Methylobacterium* significantly differed among the three sub-dams, with the highest in S523 and the lowest in S536 (Figure 2).

**FIGURE 3:**
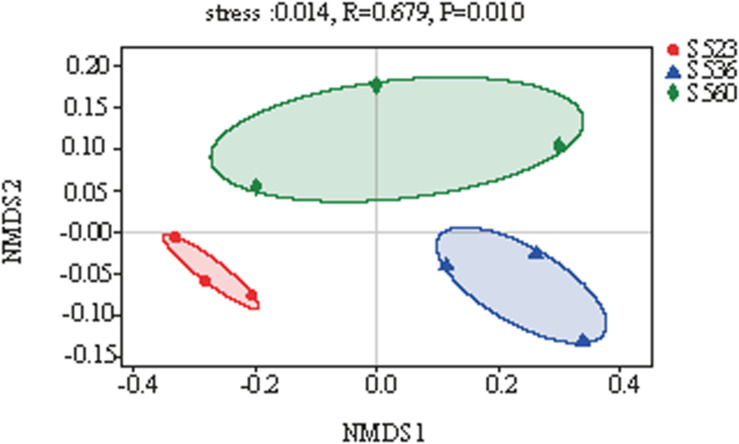
Non-metric multidimensional scaling (NMDS) of litter samples over different phytoremediation years based on the relative abundance of bacterial operational taxonomic units (OTU).

The diversity of bacteria in litter significantly differed for the different phytoremediation years. Litter bacterial community richness was lowest in S523 and highest in S536. The Shannon index of the bacterial community was highest in S523 during the early phytoremediation stage and lowest in the S536 (Table 3). As phytoremediation years progressed, litter bacterial community richness increased but gradually exhibited uneven distribution patterns. Moreover, results from NMDS analysis showed that the bacterial community composition in *B. ischaemum* litter varied significantly as phytoremediation years progressed (*P* < 0.05) (Figure 3).

**TABLE 3:** Richness and diversity indices of the litter bacterial community over different phytoremediation years.

**Sample number**	**Similarity 97%**				
	**Shannon index**	**Simpson index**	**ACE index**	**Chao index**	**Coverage**
S523	3.42*a*	0.06	160.05*b*	157.79	99.82
S536	2.07*b*	0.26	223.19*a*	199.69	99.58
S560	2.58*ab*	0.20	183.06*ab*	187.47	99.65

*Significant differences between sites (*P* < 0.05) are represented by letters (a > b).*

### Effect of Environmental Factors on Litter Bacterial Diversity

**FIGURE 4:**
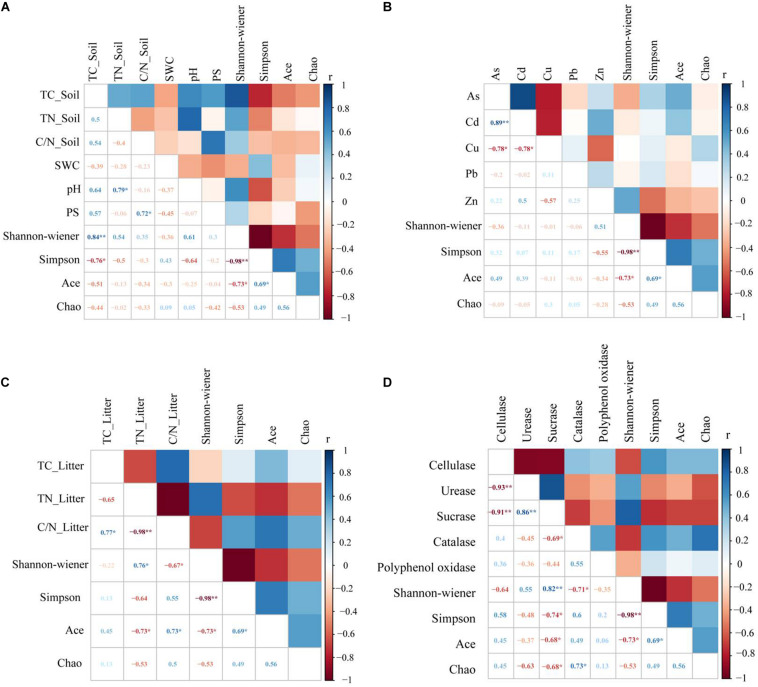
Correlations between environmental factors and bacteria diversity in litter. Environmental factors include soil properties **(A)**, heavy metals **(B)**, litter properties **(C)**, and litter enzyme activities **(D)**. Soil properties include total carbon (TC_Soil), total nitrogen (TN_Soil), the carbon and nitrogen ratio (C/N_Soil), pH, soil water content (SWC) and average particle size (PS). Litter properties include total carbon (TC_Litter), total nitrogen (TN_Litter) and the carbon and nitrogen ratio (C/N_Litter), while litter enzyme activities include cellulase, urease, sucrase, catalase and polyphenol oxidase. *Correlations are significant at a level of 0.05 (two-tailed); **correlations are significant at a level of 0.01 (two-tailed).

Litter bacterial community diversity closely correlated to soil physicochemical properties. Soil TC was positively correlated to the Shannon index and negatively correlated to the Simpson index (Figure 4A). Litter C/N was significantly negatively correlated to the Shannon index and positively correlated to the ACE index (Figure 4C). Sucrase was significantly positively correlated to the Shannon index and negatively correlated to the Simpson index, the ACE index and the Chao1 index. Catalase was negatively correlated to the Shannon index and positively correlated to the Chao1 index (Figure 4D).

### Effect of Litter and Soil Properties on the Bacterial Composition of Litter

**FIGURE 5:**
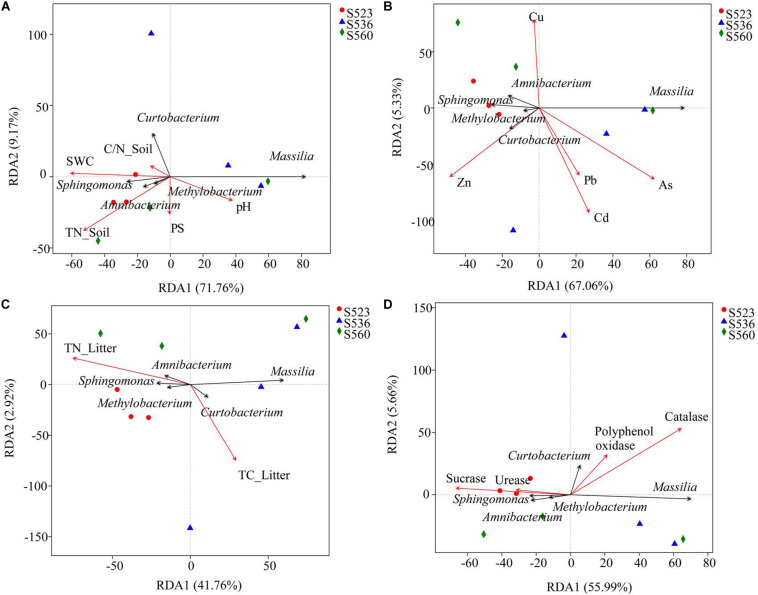
Redundancy analysis (RDA) of bacterial communities and environmental factors. Environmental factors include soil properties **(A)**, heavy metals **(B)**, litter properties **(C)**, and enzyme activities in litter **(D)**, represented by red arrows. The black arrows represent the top five bacterium at a genus level.

**FIGURE 6:**
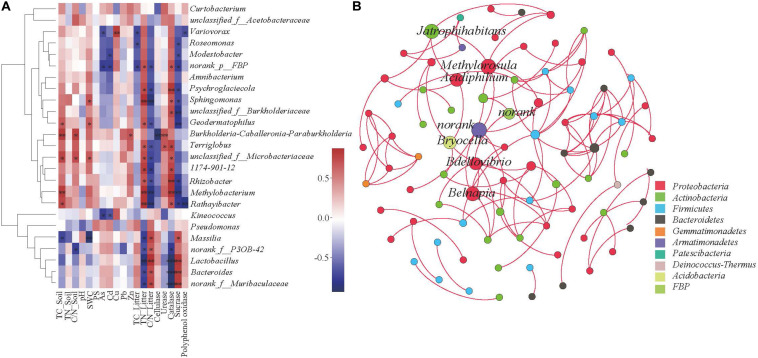
Correlation heat map of the top 25 genus **(A)** and soil and vegetation properties. The *X* and *Y* axis represent environmental factors and phyla, respectively. *R* is shown in different colors, where the right side of the legend is the color range of the different *r* values. *P < 0.05; **P < 0.01. The Co-occurrence network of bacterial taxa in litter. Nodes represent bacteria genus **(B)**, while red edges represent positive connections between species pairs, respectively.

In order to explore the driving factors that are responsible for differences in the bacterial communities of the three sub-dams, RDA was used to analyze the relationships of the dominant bacteria in *B. ischaemum* litter to soil physicochemical factors, heavy metals, physicochemical litter factors and litters enzyme activities (Figure 5). As shown in Figure 5A, RDA1 and RDA2 explained 71.76 and 9.17% of the differences in litter bacterial communities, respectively. Soil TN and SWC had the greatest influence on the bacterial community structure in litter (Figure 5A). For heavy metals, Cd and Zn had the strongest influence on the bacterial community structure in litter (Figure 5B). For the physicochemical characteristics of litter, TN in litter had a significant impact on the bacterial community structure (Figure 5C). RDA1 and RDA2 accounted for 55.99 and 5.66% of enzyme activity differences in bacterial communities, respectively (Figure 5D). Sucrase and catalase were strongly correlated to the bacterial community. Catalase activity had a significant effect on bacterial community structure (*P* < 0.05). Correlation analysis showed that *Massilia* was negatively correlated to soil TC and SWC and litter TN. Moreover, *Massilia* had a significant positive correlation to litter C/N and sucrase. *Sphingomonas* was positively correlated to litter TN and catalase while negatively correlated to litter C/N and sucrase. *Methylobacterium* was positively correlated to TC in soil and TN and catalase in litter while negatively correlated to C/N and sucrase in litter (Figure 6A). The network diagram revealed that *Methylorosula*, *Jatrophihabitans*, *Belnapia*, *Acidiphilium*, and *Bdellovibrio*, which had relatively higher betweenness centrality, played important roles in bacterial community interactions at a genus level (Figure 6B). These keystone microbes can be generally defined as those species that have a disproportionate influence on ecosystems regardless of abundance, and they are crucial in the maintenance of the stability and the function of ecosystems as well as the resistance of system disturbances.

### Functional Characteristics of Litter Bacterial Communities

**FIGURE 7:**
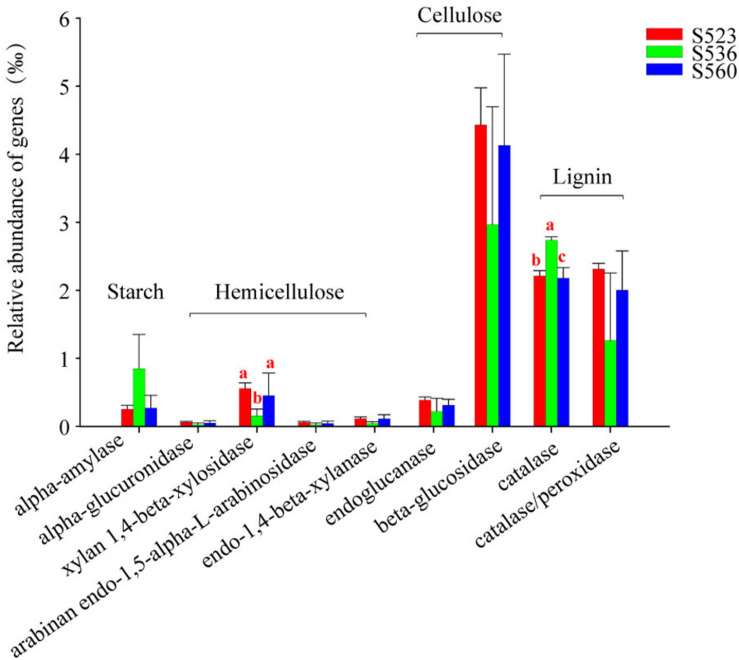
The relative abundance of functional genes associated with litter degradation within the litter bacterial community over different phytoremediation years. PICRUSt was used to predict bacteria species associated with litter degradation. Alpha-amylase was related to starch decomposition as well as other active substances. The enzymes endo-1,4-beta-xylanase, arabinan endo-1,5-alpha-L-arabinosidase, alpha-glucuronidase and xylan 1,4-beta-xylosidasewere related to hemicellulose decomposition. Endoglucanase and beta-glucosidase were involved in cellulose decomposition, and catalase/peroxidase was the main lignin-modifying enzymes. Different letters denote significant differences over different phytoremediation years (*P* < 0.05).

This study used PICRUSt to identify gene encoding enzymes associated with starch, cellulose, hemicellulose and lignin (Figure 7). Encoding gene abundance of cellulase and lignin were higher than that of amylase and hemicellulase. The abundance of cellulase and hemicellulase encoding genes was the highest in S523. Except for the encoding genes alpha-amylase and catalase, the abundance of other genes was the lowest in S536. The relative abundance of the functional encoding genes xylan 1,4-beta-xylosidase and catalase significantly differed in the three sub-dams (Figure 7). The relative abundance of functional the encoding gene catalase was the highest in S536, which was consistent with results from catalase activity (Table 2).

## Discussion

### Soil and Plant (*B. ischaemum*) Litter Characteristics

Physical and chemical soil properties are accepted as two important soil quality indices. Results from this study showed that over a progressive increase in remediation years, both soil carbon and litter nitrogen reached their maximum values during the latter phytoremediation stage (Table 1), which was consistent with previously published studies (Jia et al., 2017, 2018a; Wang et al., 2018). This result also supported our hypothesis. The main reason for this is that surface litter can add humus to the soil, effectively improving both the soil nutrient content and the soil quality (Li et al., 2015). Accumulation of Cu was the most severe during the early phytoremediation stage, while it significantly decreased during the latter phytoremediation stage. The reason for this is that *B. ischaemum* possesses a certain remediation function to extirpate heavy metals in soil, especially through its strong enrichment of Cr (Jia et al., 2018b). A previous study showed that litter from new sheathes and stems had lower nitrogen concentrations compared to older sheathes and stems (Liao et al., 2010). In our study, litter TN was relatively low in S536 compared to that in the other sub-dams (Table 1), which may have resulted from the fact that most litter in S536 had newly generated, while litter in the other sub-dams was relatively old. The decomposition process of litter by microbial activity is essentially a biochemical enzymatic decomposition process. During the early phytoremediation stage, cellulase predominated in litter (Table 2). This indicated that hemicellulose and cellulose were the two major carbon sources available to microorganisms during this stage (Schneider et al., 2012). Later, cellulase activity decreased significantly. This could have resulted from a gradual increase in litter organic matter as phytoremediation progressed. Also, the preferential use of easily decomposable cellulose by microorganisms could have led to a decrease in the utilization of cellulose, which subsequently led to a decrease in enzyme activities. On the other hand, cellulase may have derived primarily from fungi during the early litter decomposition stage. By the latter phytoremediation stage, bacteria would gradually predominate (Elisashvili et al., 2008). Catalase is an important oxidoreductase that is directly involved in both material transformation and energy flow. It is associated with organic matter content and can reflect the intensity of microbial processes (Lu et al., 2002). In this study, catalase reached its maximum value during the intermediate phytoremediation stage (Table 2), which indicated that bacteria played a relatively active role in litter decomposition during this intermediate stage.

### Litter Bacterial Community Structure and Diversity

This study found a significant difference in the relative abundance of Alphaproteobacteria and *Methylobacterium* in the three sub-dams, where S523 yielded the highest values and S536 yielded the lowest (Figure 2), which supported the hypothesis. The genus *Methylobacterium* belongs to the class Alphaproteobacteria. Taylor et al. (2012) reported 12 bacterial genera that were able to degrade sulfonated lignin, which mainly belonged to the phylum Actinobacteria and the class Alphaproteobacteria. Previous studies had shown that Proteobacteria belongs to either symbiotic or *r*-selected species, possessing clear advantages in ecological niche competitive behavior under nutrient-rich conditions. Soil and litter had the highest N content in S523. Moreover, *Methylobacterium* was significantly and positively correlated to total C in soil (TC_Soil) and total N in litter (TN_Litter) (Figure 6A). These results indicated that *Methylobacterium* is predisposed to predominate in nutrient-rich regions. The bacterial community composition in *B. ischaemum* litter exhibited significant differences during different phytoremediation years (Figure 3), which was similar to findings from Xu et al. (2020). Differences in soil physical and chemical properties during different phytoremediation years may affect litter decomposition rates, resulting in different litter degradation stages (Suseela et al., 2014). Moreover, this may be one reason that litter bacterial communities in our sample plots exhibited significant differences over different restoration years. Additionally, litter bacterial community richness was low, while bacterial community distribution was relatively uniform during the early phytoremediation stage. Although bacterial community richness in litter increased, it gradually exhibited uneven distribution patterns over progressive phytoremediation years (Table 3 and Figure 3). This may have resulted from an improvement in soil nutrients that consequently promoted an increase in plant diversity. This condition would gradually alter physical and chemical litter properties, consequently changing bacterial community richness (Urbanova et al., 2015).

### Effects of Environmental Factors on Litter Bacterial Community Characteristics

Studies have shown that litter quality effects both community decomposition and ecosystem processes (Prescott, 2010). Results from correlation analysis showed a significant correlation between soil TC and litter TN along with bacterial community diversity (Figure 4). This was a little bit different from the hypothesis. This indicated that changes in soil and litter nutrients will have an impact microbial community composition, where nutrient availability will consistently be positively correlated to microbial diversity (Yan et al., 2010). Moreover, diversity indices revealed a significant correlation between sucrase and catalase. The Shannon and Simpson indices were used to characterize soil and litter nutrients, while richness indices were best at characterizing soil enzyme activities in this copper tailings dam (Figure 4). The reason for this is that bacterial community diversity in litter is impacted by various aboveground compositional and conditional factors associated with biodiversity, while litter decomposition rates are also closely correlated to soil enzyme activities (Crumsey et al., 2015; Müller et al., 2016; Liu et al., 2017). Moreover, RDA showed that litter TN and catalase activity significantly affected bacterial community structure (Figure 5), which supported our hypothesis. Given that the behavior of different microbial populations will differ, changes in litter chemical composition and structure will result in the ecological succession of microbial populations during litter decomposition processes. Moreover, some studies have shown that soil moisture may also affect litter decomposition. In an earlier study (Lauber et al., 2009), pH was shown to be the key determining factor for bacterial composition and diversity within a localized soil layer. However, the bacterial community response to environmental factors at different spatial scales remains contentious among researchers. Within ecosystems, both bacterial community growth and activity are primarily impacted by alterations in plant litter and root exudates (Bainard et al., 2016). In our study, the effect of soil pH was not obvious, which could have been due to the alkalinity of our three sub-plots, for which no significant differences were observed. In general, litter properties were the primary factors that affected litter bacterial community characteristics in our study, particularly N content.

### Primary Driving Factors Impacting Litter Bacterial Functional Groups

In this study, *Massilia* and *Sphingomonas* had the highest overall abundance, while *Massilia* was significantly negatively correlated to soil TC and litter TN (Figure 6A). This may be due to the wide distribution of *Massilia* and its strong environmental adaptability. This genus can synthesize a variety of secondary metabolites and enzymes as well as engage in a variety of functions, such as those related to heavy metal resistance (Yang et al., 2019). Thus, we speculated *Massilia* can potentially be applied to soil remediation and improvement measures. Moreover, our study found that *Sphingomonas* was positively correlated to litter TN and negatively correlated to sucrase (Figure 6A). Using PICRUSt, we found genes encoded with cellulase, hemicellulase and lignin-modifying enzymes in litter bacterial communities, demonstrating the critical potential that bacterial communities possess in litter decomposition (Figure 7). The relative abundance of functional genes encoded with catalase was highest in S536, which could be indicative of the high heavy metal tolerance of *Sphingomonas* (Liu et al., 2017). Additionally, *Sphingomonas* predominates within alkaline environments under organic pollution, and its metabolic capacity is high (Fredrickson et al., 1995). It is important to note that functional gene distribution is only predictive of the metabolic potential and the ecological function of a bacterial community. In other words, functional gene distribution is not reflective of “real” metabolic activities and ecological functions of bacterial communities (Liang et al., 2019). Additionally, some studies have reported on the differences between functional gene abundance and gene expressions (Hollister et al., 2010; Ossola et al., 2017). Therefore, functional bacterial characteristics in litter decomposition as well as gene expression and associated regulations must be further investigated in future studies.

In brief, our results suggest that bacterial community play a crucial role in the degradation of litters and litter properties can influence the degradation of litters. However, one limitation of this work is that we did not cover the different stages of litter degradation. In the process of litter degradation, the chemistry of the organic substrate continuously changes, which will lead to the change of microbial community structure (Berg and McClaugherty, 2003). In addition, the climate change is also one of the main factors affecting litter degradation (Liang et al., 2019). Future studies will need to take into account the changes in microbial community structure and function at different degradation of the *B. ischaemum* litter. Such studies will further strengthen our understanding of the relationship between the microbial community structure and function and litter decomposition in the context of heavy metal contamination and is critically important to understand the circulation of substances in copper tailings dams.

## Data Availability Statement

The datasets presented in this study can be found in online repositories. The names of the repository/repositories and accession number(s) can be found below: https://www.ncbi.nlm.nih.gov/, PRJNA625865.

## Author Contributions

TJ conceived and designed the experiments and wrote the manuscript. YW performed the experiments. BC contributed new reagents. All authors read and approved the manuscript.

## Conflict of Interest

The authors declare that the research was conducted in the absence of any commercial or financial relationships that could be construed as a potential conflict of interest.

## Footnotes

1 www.majorbio.com
